# Glial gene networks associated with alcohol dependence

**DOI:** 10.1038/s41598-019-47454-4

**Published:** 2019-07-29

**Authors:** Emma K. Erickson, Yuri A. Blednov, R. Adron Harris, R. Dayne Mayfield

**Affiliations:** Waggoner Center for Alcohol and Addiction Research, The University of Texas at Austin, Austin, TX 78712-01095 USA

**Keywords:** Glial biology, Gene regulatory networks, Addiction

## Abstract

Chronic alcohol abuse alters the molecular structure and function of brain cells. Recent work suggests adaptations made by glial cells, such as astrocytes and microglia, regulate physiological and behavioral changes associated with addiction. Defining how alcohol dependence alters the transcriptome of different cell types is critical for developing the mechanistic hypotheses necessary for a nuanced understanding of cellular signaling in the alcohol-dependent brain. We performed RNA-sequencing on total homogenate and glial cell populations isolated from mouse prefrontal cortex (PFC) following chronic intermittent ethanol vapor exposure (CIE). Compared with total homogenate, we observed unique and robust gene expression changes in astrocytes and microglia in response to CIE. Gene co-expression network analysis revealed biological pathways and hub genes associated with CIE in astrocytes and microglia that may regulate alcohol-dependent phenotypes. Astrocyte identity and synaptic calcium signaling genes were enriched in alcohol-associated astrocyte networks, while TGF-β signaling and inflammatory response genes were disrupted by CIE treatment in microglia gene networks. Genes related to innate immune signaling, specifically interferon pathways, were consistently up-regulated across CIE-exposed astrocytes, microglia, and total homogenate PFC tissue. This study illuminates the cell-specific effects of chronic alcohol exposure and provides novel molecular targets for studying alcohol dependence.

## Introduction

Glial cells, such as astrocytes and microglia, are important for proper brain development and function^1^. Glial dysfunction is thought to be involved in neurological and psychiatric disorders, including depression^2–5^, Alzheimer’s disease^6,7^, and drug addiction^8^ including alcohol use disorders (AUDs). Alcohol exposure changes astrocyte and microglia morphology and phenotype^9–13^, implicating glial cells as a target of alcohol. Furthermore, transcriptome studies of human alcoholic and chronic alcohol-exposed rodent brains identified gene expression changes that were specifically related to glial function^14–17^.

Functional consequences of sustained gene expression changes in astrocytes and microglia may regulate behavioral traits relevant to AUDs. For example, pharmacological and chemogenetic manipulation of astrocyte function altered the motivation to self-administer drugs of abuse^13,18–21^. A cell type-specific depletion study showed a role for microglia in alcohol’s effects on neuroimmune gene expression^22^, and null mutant mice lacking different immune genes demonstrated potential roles for neuroimmune signaling in alcohol consumption^23,24^. In addition, cell-specific transgenic mouse models showed that glial-derived cytokines modulate drug-induced behaviors^25–27^. Thus, there is consistent support that alcohol-induced gene expression changes in glia regulate neuroadaptations and behaviors associated with AUD.

As the immune cells of the CNS, astrocytes and microglia express many of the same genes and pathways, especially those related to neuroimmune receptors and signaling components^28,29^. It is difficult to distinguish cell type-specificity of “glial-associated” gene expression changes using bulk RNA-sequencing (whole tissue analysis utilizing RNA from all brain cells) methodology. This is further complicated by the fact that astrocytes and microglia also express genes often assumed to be explicitly neuronal^30^. In addition, activation of the same receptor signaling pathway in distinct cell types can lead to diverse physiological and behavioral consequences^31,32^. Recent studies showed that chronic, voluntary alcohol consumption produces distinct changes in gene expression in astrocytes and microglia that are mostly not detected in total homogenate^33,34^. Delineating these cellular effects is a crucial step towards understanding the complex molecular brain adaptations associated with AUDs.

Here, we establish the astrocyte- and microglia- specific transcriptome alterations in mouse prefrontal cortex (PFC) following chronic intermittent ethanol vapor exposure (CIE). CIE utilizes passive exposure to alcohol vapor to yield blood alcohol concentrations (BAC) that are difficult to achieve using traditional animal models of voluntary alcohol self-administration^35^. Periods of high BAC alternated with intermittent periods of withdrawal reliably leads to escalated voluntary alcohol consumption and severe symptoms of dependence in rodents^36^. In addition, the neurobiological and behavioral adaptations found in humans with AUD are paralleled in studies of CIE-exposed rodents^37,38^, and clinically effective medications can reduce CIE-induced increases in alcohol consumption^39^. Using RNA-sequencing, we profiled transcriptomes of astrocytes and microglia (as well as total homogenate) from the PFC of CIE-exposed mice. Differential expression analysis revealed marked gene expression changes in astrocytes and microglia isolated from alcohol-dependent mice; however, there was little overlap with CIE-induced changes observed in total homogenate samples. We used weighted gene co-expression network analysis (WGCNA) to identify unique co-expressing networks of genes altered in CIE-treated in astrocytes and microglia. From these networks, we discovered novel cell type-specific genes that may be involved in the pathophysiology of alcohol dependence.

## Materials and Methods

### Mice

Adult (8 weeks) male C57BL/6J mice were purchased from The Jackson Laboratory (Bar Harbor, ME). Mice were housed in the Animal Resource Center at The University of Texas at Austin with 12 h light/dark cycles and maintained on a standard laboratory diet and water *ad libitum*. All experiments were approved by The University of Texas at Austin Institute for Animal Care and Use Committee and conducted in accordance with NIH guidelines regarding use of animals in research.

### Chronic intermittent ethanol vapor exposure (CIE)

**Figure 1:**
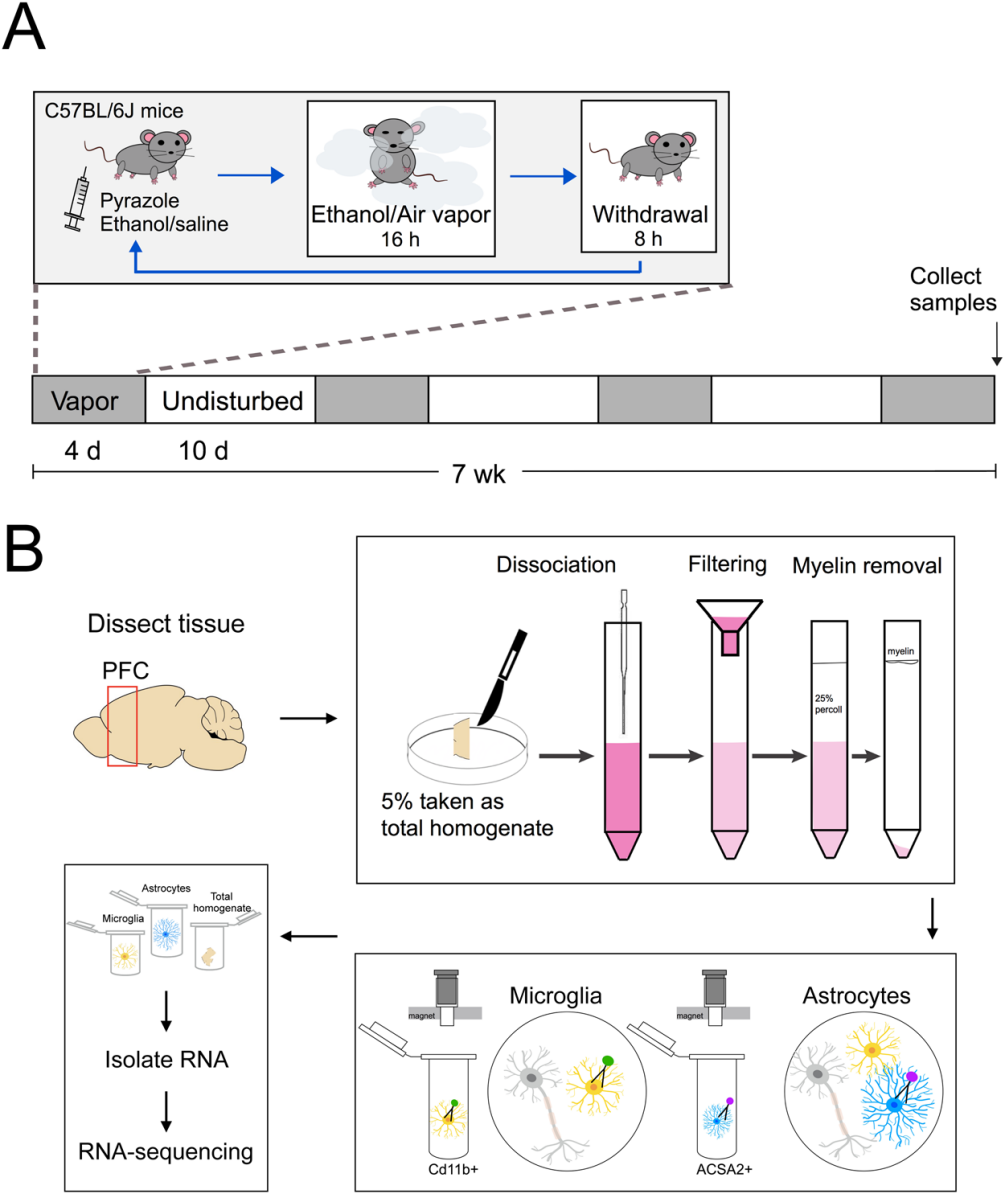
Schematic of alcohol exposure and cell isolation methods. (**A**) Chronic intermittent ethanol vapor procedure. (**B**) Cell type isolation procedure.

Briefly, ethanol was volatilized, mixed with fresh air and delivered to Plexiglas inhalation chambers at a rate of 10 L/min^40^. Alcohol concentrations in the chamber were maintained at 15–20 mg/L air. Before entering the chambers, mice (12 alcohol + 12 control = 24 total) were administered alcohol (1.6 g/kg; 8% w/v) and the alcohol dehydrogenase inhibitor pyrazole (1 mmol/kg) in a volume of 20 ml/kg body weight to help stabilize and maintain BACs. Control mice received saline, pyrazole and fresh air (also in Plexiglas inhalation chambers). Animals were left in the chamber for 16 h/day during 4 weekly cycles, each cycle alternated with weeks in between in which mice were left undisturbed (Fig. 1A). Immediately following their last vapor treatment, blood samples were obtained using retro-orbital bleeding. Samples were collected into capillary tubes and centrifuged for 6 min at 3100 g in a Haematospin 1400 centrifuge (Analox Instruments, Lunenburg, MA). Plasma samples were stored at −20 °C until BACs were determined in 5 μl aliquots using an AM1 Alcohol Analyzer (Analox Instruments). BACs were determined using commercially available reagents according to the manufacturer’s instructions. BACs averaged 171 ± 33 mg/dl (*n* = 10).

### Cell enrichment and RNA isolation

Mice were anaesthetized with 3% isoflurane and immediately perfused with phosphate-buffered saline (PBS). The PFC was dissected by removing the olfactory bulbs, then cutting the foremost 2 mm of the cortex from each side at an approximate 50° angle from the midline, as previously described^16^. Tissue was minced and dissociated into a single cell suspension using the Neural Tissue Dissociation Kit with Papain (Miltenyi Biotec, Bergish Gladbach, Germany). Myelin was removed by centrifugation in a 25% Percoll gradient. Astrocyte enrichment was performed using astrocyte cell surface antigen-2 (ACSA2) magnetic MicroBeads (Miltenyi Biotec). Microglia were subsequently isolated from the ACSA2- cell fraction using Cd11b magnetic MicroBeads (Miltenyi Biotec) (Fig. 1B). This purification technique was selected based on previous successful studies in the adult rodent brain^33,34,41–44^. RNA was extracted using the RNeasy Micro Kit (Qiagen, Hilden, Germany) and examined on the Bioanalyzer (Agilent Technologies, Santa Clara, CA) for quality and quantity. Total RNA concentrations for astrocytes and microglia averaged 2190 pg/μl and 369 pg/μl, respectively. RNA integrity number (RIN) scores for astrocyte total RNA ranged from 7.2–9.4, while RIN scores for microglia total RNA ranged from 5.7–9.6.

### RNA sequencing, quality control and read processing

RNA was submitted to the Genomic Sequencing and Analysis Facility at The University of Texas at Austin for mRNA selection using the MicroPoly(A) Purist Kit (Life Technologies, Carlsbad, CA) and library preparation using NEBNext Module Components (New England Biolabs, Ipswich, MA). Four microglia samples were excluded due to low RNA quantity (>1 ng per sample). Samples were sequenced on the Illumina Hi-Seq 4000 at a depth of 20 million paired-end reads (150 bases/read). Read quality was assessed using FASTQC (version 0.11.5)^45^. Adapters were removed with Cutadapt (version 1.8) and were mapped to the mouse genome (UCSC mm 10) using STAR (version 2.5.0a)^46^. Duplicate reads were filtered from sorted BAM files using Picard’s MarkDuplicates tool (version 1.141). Raw counts were quantified using HTSeq (version 0.6.1p1)^47^. Three samples were determined to be outliers using principal component analysis and hierarchical clustering analysis and were excluded. The final analyses included 17 samples (8 control + 9 alcohol-treated mice) for each cell type. Raw and processed sequencing data from this study have been deposited to the Gene Expression Omnibus under the accession number GSE128561.

### Bioinformatics analysis

Differentially expressed genes were identified using the Bioconductor package DESeq2^48^. We used a significance threshold of an adjusted p-value of 0.05, calculated from the Benjamini-Hochberg false discovery rate (FDR), for differential expression analysis. Functional enrichment of differentially expressed genes in each cell type was identified using the online tool Enrichr^49^. WGCNA was used to identify groups of co-expressing genes in astrocytes and microglia^50^. WGCNA inputs were log2-transformed normalized counts for each sample, excluding genes containing 0 counts in any of the samples. WGCNA was performed on astrocyte and microglia samples independently using the *R* package^50^.

The general framework of WGCNA has been previously described^51^. Briefly, we constructed a signed adjacency matrix by calculating Pearson correlations for all pairs of genes. To emphasize strong correlations on an exponential scale, we raised the adjacency to power β. We chose a power of β = 17 for both cell types so the resulting networks exhibited approximate scale-free topology (scale free topology fit = 0.85). To identify gene modules, all genes were hierarchically clustered based on connection strength determined using a topological overlap dissimilarity calculation. Resulting gene dendrograms were used for module detection using the dynamic tree cut method (minimum module size = 100). Quality statistics were calculated for identified gene modules in each cell type using modulePreservation^52^ (see Supplementary Information). To determine module-trait relationships, Pearson correlations were calculated for module eigengene expression with CIE treatment status and BAC. BAC correlations were calculated using only the CIE-treated samples. Resulting p-values from module-trait correlations were adjusted for multiple comparisons using an FDR threshold of 0.05. To functionally characterize modules, all genes belonging to a module were submitted to Enrichr for biological process gene ontology and pathway analysis. Module visualizations were created with the top 50 edges (based on topological overlap connectivity) of each module using Gephi (version 0.9.1)^53^. We identified hub genes for each module by examining correlation with module eigengene and within-module connectivity.

### Immunohistochemistry

A separate group of CIE-exposed mice were used for protein expression analysis. Mice were anaesthetized with isoflurane and perfused with PBS and 4% paraformaldehyde (PFA). Brains were post-fixed in PFA for 24 h, then cryoprotected in 20% sucrose solution for 24 h. Brains were frozen in optimal cutting temperature and stored at −80 °C until slicing. Brains were sliced (30 μm coronal sections), permeabilized in 0.1% Triton-X for 10 min, and blocked in 10% donkey serum for 1 h at RT. The sections were then incubated overnight at 4 °C in primary antibody (rabbit anti-CEBPD 1:500, Rockland Immunochemical, Pottstown, PA, Catalog #10800-764). Following three washes in PBS, sections were incubated in secondary antibody (donkey anti-rabbit 488, Thermo Fisher Scientific, Rockford, IL) for 2 h at RT, mounted in 0.2% gelatin, dehydrated, and cover slipped with mounting medium including DAPI (Vector Labs, Burlingame, CA). Slides were visualized using a Zeiss Axiovert 200 M fluorescent light microscope (Zeiss, Thornwood, NY). Bilateral images of the PFC (Bregma +2.8 to +2.24) were captured using a 20x objective. Images were analyzed using ImageJ (version 1.50i). CEBPD+ cells were quantified in medial regions of the PFC in two regions of interest per section (box, 700 × 700 μm). Cell counts were averaged between two sections per animal (*n* = 4).

## Results and Discussion

### Chronic intermittent ethanol elicits robust changes in astrocyte and microglia gene expression

**Figure 2:**
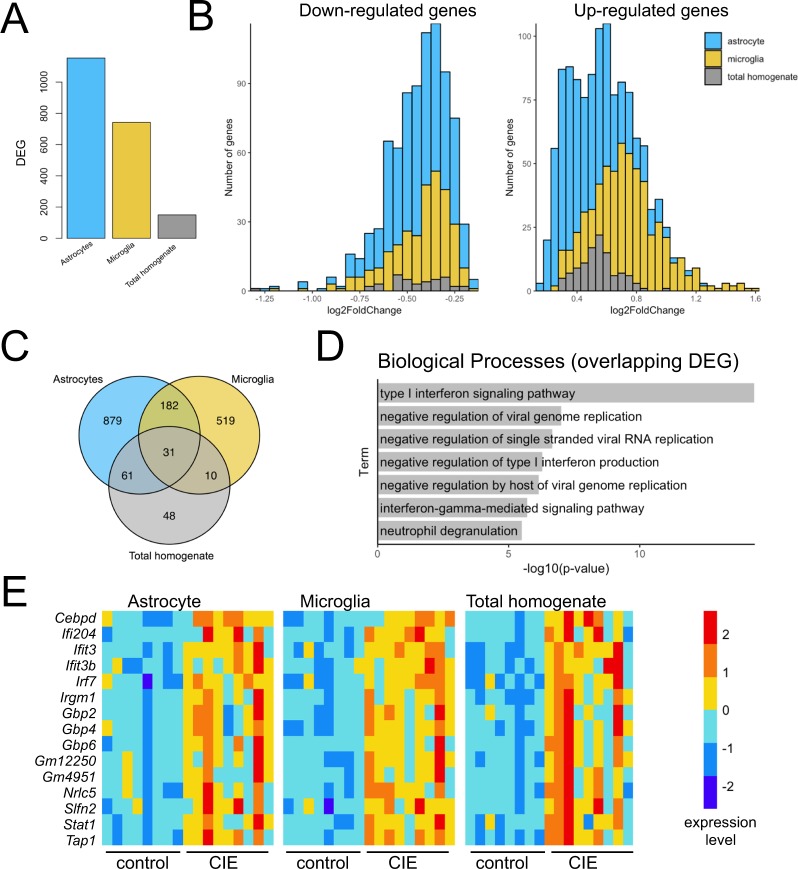
Differential expression comparison by cell type. (**A**) Number of CIE-induced differentially expressed genes (DEG) (p_adj_ < 0.05) identified in astrocytes, microglia, and total homogenate. (**B**) Histograms plotting magnitude of fold changes for up- and down-regulated DEG identified in each tissue type. (**C**) Overlap of DEG between astrocytes, microglia, and total homogenate. (**D**) Biological process enrichment results of overlapping DEG across all cell types. (**E**) Heat maps displaying expression levels of interferon-related DEG across control and CIE-treated samples for astrocytes, microglia, and total homogenate. Color indicates expression level (regularized log transformed gene counts).

Differential expression analysis revealed 1153 and 742 differentially expressed genes following CIE (adjusted *p* < 0.05) in astrocytes and microglia, respectively (Supplementary Table 1). In contrast, only 150 genes were differentially expressed in total homogenate samples (Fig. 2A). Thus, cell type-specific analysis detected a significantly greater number of differentially expressed genes (~13x) compared with total homogenate preparations. Up-regulated genes found in isolated microglial preparations displayed larger magnitude fold changes compared with those in total homogenate and astrocytes (Fig. 2B). Between microglia and astrocytes, 213 differentially expressed genes were shared, while fewer were shared between total homogenate and either astrocytes (91 genes) or microglia (40 genes) (Fig. 2C). A total of 31 genes were differentially expressed in all three cellular preparations. Enrichment analysis demonstrated that overlapping changes in astrocytes, microglia, and total homogenate were consistently involved in type I interferon signaling (Fig. 2D). Interferons are produced following activation of toll-like receptors (TLRs), which are pattern-recognition receptors that regulate the initiation of innate immune signaling cascades in brain^54^. Among the interferon-related genes up-regulated in all three preparations following CIE exposure were *Ifi204* (involved in interferon production), *Irgm1*, *Ifit3*, *Ifit3b*, *Gbp2*, *Gbp4*, *Gbp6* (induced by interferon signaling), *Irf7* (interferon regulatory factor), *Stat1*, *Cebpb*, and *Cebpd* (activated by interferon signaling) (Fig. 2E). It is well established that alcohol activates immune gene expression in brain^12,55,56^, and neuroimmune molecules modulate drinking behavior^24,57–60^. Our data suggest that CIE exposure initiates neuroinflammatory response involving type I interferon signaling that is generalized across astrocytes, microglia, and possibly neurons, as a major fraction of the genes expressed in the total homogenate are neuronal.

### Glial gene networks associated with CIE: Overlapping changes between cell types

**Figure 3:**
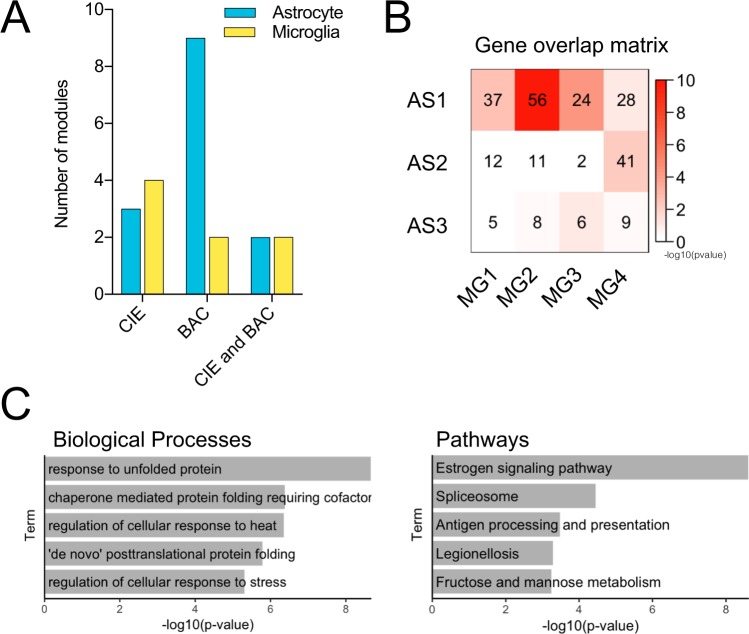
Weighted gene co-expression network analysis: similarities between astrocyte and microglia trait-associated modules. (**A**) Number of modules found in astrocyte or microglia gene networks that significantly correlated with CIE treatment, BAC, or both. (**B**) Gene overlap matrix demonstrating the number of genes that overlapped between each pair of CIE treatment-associated modules in astrocytes (AS1-3) and microglia (MG1-4). Significance of overlap (−log10[p-value]) as measured by a Fisher’s exact test is indicated by color. (**C**) Biological process and pathway enrichment analysis for overlapping genes identified in CIE-associated modules in both cell types.

Using WGCNA to identify networks of co-regulated genes, we detected 23 gene modules in astrocytes and 19 in microglia. We next investigated the relationship between module eigengene expression and specific traits, treatment group and BAC (Supplementary Table 2). Of the 23 astrocyte modules, nine correlated with BAC, three were associated with CIE treatment, and two were associated with both CIE and BAC. In microglia, four modules were associated with CIE and two modules correlated with both CIE and BAC (Fig. 3A). We also determined the overlap of genes between modules associated with CIE treatment in different cell types. Significant overlap was observed between a subset of treatment-associated modules (Fig. 3B). For example, AS1 shared several genes with microglia modules, notably MG2. Genes common to both astrocyte and microglia CIE-associated modules were enriched for stress response processes involving protein folding and estrogen signaling (Fig. 3C). These results were driven by overlapping differentially expressed heat shock factor (HSF)-induced genes (*Hsp90ab1*, *Hspa1a*, and *Hspd1*), which can initiate protective responses to stress. This indicates that alcohol may induce common stress responses in different cell types. Indeed, HSF-induced genes have been implicated in alcohol-mediated gene expression changes in both neurons and astrocytes^61,62^. Estrogen signaling, which is regulated by heat shock proteins, has neuroprotective and anti-inflammatory functions dependent on receptor expression in astrocytes^63,64^. Estrogen signaling also regulates microglial activation^65^. Our findings indicate that glia respond to chronic alcohol with alterations in common stress pathways coordinately expressed within cell type-specific networks. Conversely, some astrocyte modules showed little to no overlap with treatment-associated microglial modules, suggesting alcohol-induced changes in unique sets of genes depending on cell type.

### Astrocyte gene networks associated with CIE

Enrichment analysis of CIE-associated gene networks indicate a variety of astrocyte signaling pathways may be perturbed in the PFC of alcohol-dependent mice. We focused on two CIE-associated astrocyte modules (AS1 and AS3) for further discussion due to their statistical significance and overall biological relevance for astrocyte function.

**Figure 4:**
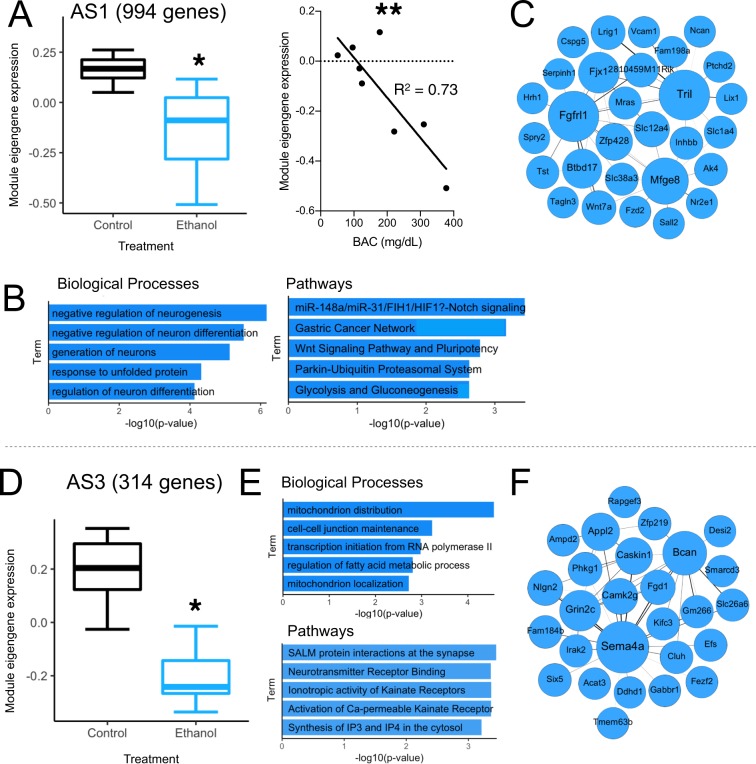
Astrocyte-specific gene networks regulated by CIE treatment. (**A**–**C**) Pertain to AS1 and (**D**–**F**) pertain to AS3. (**A**,**D**) Module eigengene expression of both modules was down-regulated with CIE treatment. Module eigengene expression of AS1 was significantly correlated with blood alcohol concentration (BAC) (Pearson correlation). (**B**,**E**) Biological process and pathway enrichment analysis of genes within astrocyte modules. (**C**,**F**) Top 50 gene correlations within astrocyte modules. Circle size corresponds to number of connections while line thickness corresponds to connection strength (correlation between gene expression level). **p-value < 0.01, *p-value < 0.05.

AS1 (994 genes) was negatively associated with CIE and BAC (Fig. 4A). Functional enrichment analysis revealed processes related to nervous system development, including “Negative regulation of neurogenesis”, and developmental pathways such as Notch and Wnt signaling (Fig. 4B). Notch- and Wnt- related genes have been previously shown to be altered by chronic alcohol exposure^15,66^ and to regulate alcohol-induced synaptic remodeling^67^. Recent studies suggest they may be important for controlling mature astrocyte function and plasticity^68,69^. Changes in Notch or Wnt signaling may signify altered astrocyte identity, as both pathways have both been implicated in astrocyte reactivity^70–72^, a general phenomenon indicating broad changes in molecular phenotype and function, occurring in response to injury or disease^73^. Neuroinflammation is often a key driver of astrocyte reactivity. Indeed, we identified highly connected hub genes within AS1 involved in inflammation, including *Mfge8* and *Tril* (Fig. 4C). *Mfge8* encodes milk fat globule-EGF factor 8, a secreted protein that may mark specific astrocyte subpopulations or reactivity states in the cortex^74^. While *Mfge8* regulates apoptosis, inflammation, and phagocytosis^75–77^, current knowledge of its role in astrocyte function is scarce. Previously found to be down-regulated in human alcoholic frontal cortex^78^, *Mfge8* may be a novel astrocytic target for alcohol use disorders. Another highly connected hub gene, *Tril* (TLR4 interactor with leucine-rich repeats), is an astrocyte-enriched gene that facilitates TLR-regulated immune activation specifically in brain^79,80^. A strong link exists between alcohol exposure and neuroinflammation mediated through TLR activation^12,81,82^, and many components of TLR signaling cascades have been interrogated genetically for their role in alcohol-related behavioral phenotypes^55^. *Tril* has not yet been explored in this manner, but our results suggest it may be another important alcohol-responsive neuroimmune gene in astrocytes. Overall, results from the AS1 module suggest a relationship between classical neurodevelopmental signaling pathways and neuroimmune dysfunction in astrocytes isolated from CIE-exposed mice.

AS3 (314 genes) was also negatively associated with CIE treatment (Fig. 4D), but not significantly correlated with BAC. AS3 was functionally enriched with gene categories such as “Protein interactions at the synapse”, “Neurotransmitter receptor binding”, and “Synthesis of IP3 and IP4 in the cytosol” (Fig. 4E). These categories include genes related to astrocyte-neuronal communication, specifically synaptic GPCR signaling leading to intracellular Ca^2+^ elevations. Ca^2+^ signaling mediates a plethora of astrocyte functions, including release of gliotransmitters like ATP, glutamate, and D-serine, all of which can modulate neuronal activity^83^. In primary astrocyte cultures, alcohol interferes with astrocyte Ca^2+^ responses to specific stimuli^84^, and our findings suggest chronic alcohol exposure may also impair astrocyte Ca^2+^ signaling *in vivo*, potentially compromising glial-neuronal interactions in mouse PFC. Mitochondrion distribution, one of the top significantly enriched biological processes found here, is implicated in the regulation of Ca^2+^ responses at perisynaptic processes^85^. Hub genes in AS3 included genes coding for membrane-bound receptors often found at synapses (*Sema4a*, *Grin2c*, and *Gabbr1*), as well as structural components of synapses (*Bcan*, *Caskin1*, *Nlgn2*) (Fig. 4F). Several of these genes have been implicated in astrocyte responses to neurotransmitters and Ca^2+^ elevations. For example, expression of *Sema4a* is altered in astrocytes in response to inflammatory mediators that disrupt astrocyte Ca^2+^ signaling^86^. *Grin2c* encodes a subunit of the NMDA receptor known to elicit Ca^2+^ elevations in astrocytes and influence neuron-to-glia signaling^87,88^. *Gabbr1* encodes a GABA_B_ receptor expressed on astrocytes that promotes Ca^2+^-induced release of gliotransmitters^89–91^. Interestingly, the GABA_B_ receptor ligand baclofen has been suggested as a treatment for AUD, but its molecular mechanism is not completely understood^92^. GPCR agonists (*e*.*g*. baclofen) intended to target neuronal signaling may affect astrocyte function in unknown ways, because astrocytes express many of the same receptors as neurons, but may signal though unique mechanisms to produce distinct functional consequences^93–96^. Overall, AS3 enrichment results indicate dysfunction in synaptic signaling involving astrocyte GPCR activation and subsequent Ca^2+^ signaling. Together, these astrocyte-specific networks highlight the potential importance of neuroimmune genes and neurotransmitter receptors in astrocytes that have not been explored in alcohol-dependent brain, providing a framework for future targeted studies.

### Microglial gene networks associated with CIE

Two microglia modules (MG1 and MG2) were significantly correlated with both CIE treatment and BAC. We examined these in more detail given their relevance for microglial function.

**Figure 5:**
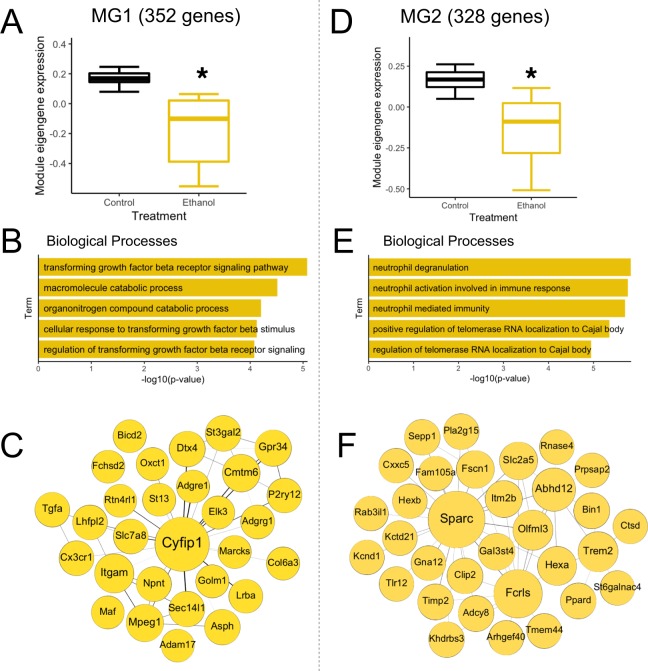
Microglia-specific gene networks regulated by CIE treatment. (**A**) Module eigengene expression of MG1 was significantly down-regulated in CIE-treated microglia. (**B**) Biological process enrichment analysis of MG1 genes. (**C**) Top 50 gene correlations within MG1. Circle size corresponds to number of connections while line thickness corresponds to connection strength (correlation between gene expression level). (**D**) Module eigengene expression of MG2 was significantly down-regulated in CIE-treated microglia. (**E**) Biological process enrichment analysis of MG2 genes. (**F**) Top 50 gene correlations within MG2. *p-value < 0.05.

MG1 (352 genes) was negatively correlated with CIE treatment and BAC (Fig. 5A). This network was enriched with genes related to transforming growth factor beta receptor (TGF-βR) signaling (Fig. 5B), including *Tgfbrap1*, *Smad3*, *Smad5*, and *Itgb5*. TGF-β1 is a crucial modulator of microglia homeostasis^97,98^. TGF-β1 modulates gene expression through activation of *Smad3*, is induced by an inflammatory event and exerts neuroprotective effects, ultimately working to limit the spread of neuroinflammation^99,100^. Other microglia-specific genes known to be involved in TGF-β signaling or homeostatic maintenance were highly connected within MG1 (e.g., *Cx3cr1* and *P2ry12*) (Fig. 5C). *Cx3cr1* encodes CX3C chemokine receptor 1, which is activated by the neuronal chemokine fractalkine^101,102^. Fractalkine signaling is important in many normal microglial functions, such as regulation of synaptic plasticity and brain connectivity, migration, and constant immune surveillance^103^. Mice lacking microglial *Cx3cr1* show decreased TGF-β1 signaling and impaired functional recovery following brain injury^104^. The gene for P2RY12 (purinergic receptor P2Y, G-protein coupled, 12) was also highly connected within MG1. P2RY12 is a marker of mature microglia, helps direct the migration of motile cell processes towards sites of injury or inflammation within the CNS^105^, and may promote a neuroprotective microglial phenotype^106,107^. Another hub gene, *Cyfip1*, which encodes cytoplasmic FMRP interacting protein 1, is less well characterized regarding microglial function. However, variants of this gene have been linked to a variety of developmental brain disorders^108^, and recent work suggests haploinsufficiency of *Cyfip1* disrupts neurogenesis via a microglia-specific mechanism^109^. The negative correlation of MG1 gene expression with CIE and BAC suggests that CIE exposure produces dysregulation of key genes and pathways relevant for microglia homeostasis and immune surveillance.

MG2 (328 genes) was negatively correlated with CIE and BAC and was enriched with genes related to neutrophil degranulation (Fig. 5E). Highly connected genes in this module included *Trem2*, *Sparc*, and *Abhd12*, all of which have established roles in microglial function (Fig. 5F). Polymorphisms in *TREM2* are associated with increased risk of Alzheimer’s disease in humans^110^ and its expression is involved in microglial function and survival in mouse models^111,112^. In addition, *Trem2* expression is negatively associated with predisposition for alcohol consumption in mice^113^. *Sparc* regulates distribution and branching of microglia as well as activation and recovery following injury^114^, and also controls synapse formation and elimination^115^. *Abhd12* encodes an enzyme that regulates hydrolysis of the lipid transmitter 2-arachidonoyl glycerol, as well as the metabolism of other immunomodulatory signaling lipids^116^. Disruption of *Abhd12* activity could lead to the accumulation of proinflammatory lipids that promote abnormal microglial function^117^. Overall, the association of these genes with CIE treatment and BAC suggests an impaired microglial inflammatory response following exposure to chronic alcohol. The majority of hub genes in the top microglial networks associated with CIE have not been studied in relation to alcohol-induced neuroinflammation or alcohol behaviors, and these genes provide novel microglial targets for understanding cell type-specific responses in AUD.

### CIE-associated glial gene networks contain genes relevant to alcohol-related behaviors

We compared astrocyte and microglia gene modules significantly associated with CIE treatment and BAC with genes previously shown to influence alcohol behaviors in mice^118^. Several genes within these alcohol-responsive modules have been linked to changes in alcohol consumption or alcohol-sensitive behaviors. For example, AS1 contained the metabotropic glutamate receptor *Grm5*, which regulates alcohol preference and other related behaviors^119^. AS1 also contained *Chrna7*, a nicotinic acetylcholine receptor subunit expressed in astrocytes^120^ shown to modulate alcohol consumption^121^. In addition, the astrocyte glutamate transporter gene *Slc1a3* was a hub gene in a module that was negatively correlated with BAC. Dysregulation of astrocyte glutamate uptake has been implicated in alcohol reward and withdrawal^19,21^, and *Slc1a3* knockout mice show reduced voluntary alcohol consumption^122^. MG2, a microglial network negatively associated with CIE treatment, includes the gene *Ctsf*, a.k.a. Cathepsin F, which regulates alcohol consumption and preference in mice^24^. This module also contains *Slc29a1*, which encodes an adenosine transporter also shown to regulate alcohol consumption^123^. Through association with established genes known to be involved in alcohol behaviors, these newly identified gene networks and hub genes may significantly impact alcohol-related behaviors.

### Cell-specific transcriptome changes across alcohol exposure models

The CIE protocol described in the present study is routinely implemented to mimic the high BACs regularly achieved in humans, and results in physiological neuroadaptations that facilitate severe alcohol dependence^124,125^. Another commonly used animal model of alcohol abuse is a voluntary, chronic every-other-day drinking paradigm (EOD), which causes escalated alcohol consumption over time, but not severe alcohol dependence^126,127^. We have previously examined transcriptome responses in cortical astrocyte and microglia populations isolated from mice following the EOD procedure^33,34^. To determine the effects of different alcohol exposure procedures on perturbations in glial transcriptomes, we compared differentially expressed genes identified in the present study with our previous results, (using a nominal p-value cutoff of 0.05 for differential expression). We observed substantial variation in glial transcriptome responses. Compared to EOD, CIE treatment led to several hundred more differentially expressed genes in PFC astrocytes and microglia. Despite this difference, a significant number of the alcohol-responsive genes were observed in both alcohol paradigms. In astrocytes, there were 194 overlapping genes that were differentially expressed in the present CIE study and after EOD drinking (hypergeometric p = 3.74e-19)^33^. For differentially expressed microglia genes, there were 231 overlapping genes in the present study and after EOD drinking (hypergeometric p = 5.88e-46)^34^. Astrocyte genes that were differentially expressed in response to both EOD and CIE were enriched with synaptic vesicle signaling, amino acid transmembrane transporter activity, and mitochondrial distribution. Conserved microglia responses were enriched with immune-related genes, specifically TGF-β signaling. Thus, two different chronic alcohol exposures, using intermittent (EOD) voluntary drinking or intermittent passive exposure to alcohol vapor, elicited overlapping molecular changes in cortical glia. These overlapping changes could play a role in the transition from regulated to excessive drinking. While we did not measure alcohol drinking in these mice, when combined with free-choice drinking, repeated cycles of CIE vapor robustly contributes to escalation of alcohol consumption over time^35,126^. Importantly, CIE exposure itself is useful for modeling several aspects of alcohol dependence, including withdrawal, tolerance, learning and memory disturbances, altered stress signaling and reward function^36^. Alcohol-responsive genes identified only in glia from CIE-exposed mice may regulate these CIE-induced phenotypes. Further testing will be required to determine the roles that specific glial genes have in shaping neurobiology of AUD.

### Immunohistochemistry analysis of a gene differentially expressed in both glial cell types

A consistent finding across cell types was that CIE treatment led to increased expression of genes associated with neuroinflammation. For example, *Cebpd* was up-regulated in astrocytes, microglia, and the total homogenate from alcohol-dependent mice (Fig. 2D). *Cebpd* encodes a member of the CCAAT-enhancer binding protein (C/EBP) family of transcription factors, which regulate immune responses and other biological processes. *Cebpd* transcription can be induced by a variety of inflammatory signals^128^. CEBPD is highly expressed in activated glia and is known to regulate glial pro-inflammatory gene expression^129^, contribute to oxidative stress^130^, and the progression of neurodegenerative disease^131^. Because of its broad regulatory potential and the absence of previous studies investigating CEBPD in the alcohol-dependent brain, we studied its expression in the PFC of CIE-treated mice at the protein level.

**Figure 6:**
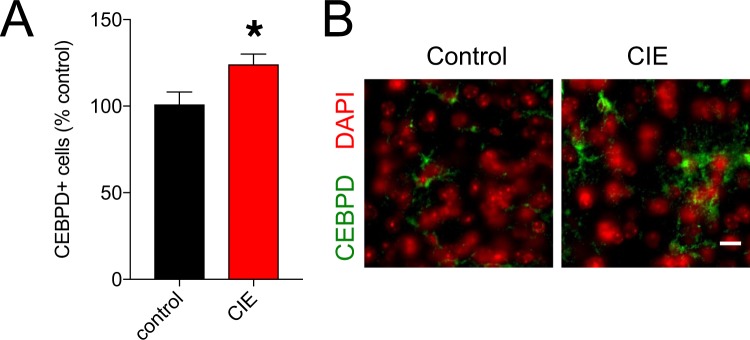
Immunostaining of CEBPD in PFC tissue from control or CIE-treated mice. (**A**) Quantification of CEBPD+ cells. (**B**) Representative images of CEBPD staining in PFC. Scale bar = 20 μm.

Immunohistochemistry revealed CIE-treated mice had an increased number of CEBPD+ cells in the PFC (Fig. 6). This suggests more cells have the capacity for CEBPD signaling in alcohol-dependent brains, perhaps the result of an increasingly inflammatory microenvironment. This finding is consistent with the increased gene expression related to interferon signaling found across glia, and further supports the hypothesis that repeated exposure to high concentrations of alcohol induces a neuroinflammatory state^132^. In voluntarily drinking, non-dependent mice, increased expression of *Cebpd* was observed in isolated astrocytes, but not in microglia or total homogenate^33,34^. *Cebpd* expression in different cell types may thus depend on the alcohol exposure model and BAC. Considering the role for CEBPD in learning and memory^133,134^, it is of further interest to study its relevance in the behaviors associated with the transition to alcohol dependence.

## Conclusions

The transcriptomes of astrocytes and microglia in the mouse PFC were strongly perturbed by a chronic alcohol exposure paradigm that induces alcohol dependence. Most of the cell type-specific changes in gene expression were not observed in total homogenate, which underscores the importance of using distinct cellular populations to characterize molecular responses to alcohol. Astrocytes and microglia exhibited mostly non-overlapping gene network responses to alcohol, which may reflect their distinct functions in maintaining central nervous system homeostasis. Altered gene networks in astrocytes suggested that there are important changes in astrocyte identity and synaptic Ca^2+^ signaling in alcohol-dependent mouse brain. In microglia, homeostatic functions mediated by TGF-β signaling, as well as genes involved in inflammatory response, were disrupted by CIE treatment. Several of the genes within these networks have been identified in previous alcohol-related studies, some of which were causally linked to changes in drinking behavior. Shared differentially expressed genes between astrocytes and microglia indicate that common cellular stress mechanisms are altered in glial cells in response to chronic alcohol. Across both glial cell types and the total homogenate, changes in a subset of genes consistent with neuroimmune activation were also observed. Immunohistochemistry results support the hypothesis of alcohol-induced neuroinflammation and pose interesting questions concerning the role of CEBPD signaling in alcohol dependence. Furthermore, comparison of our datasets with previously published cell-specific transcriptome profiles unveiled molecular similarities as well as disparities between different models of chronic alcohol exposure. Gene expression changes identified in response to both CIE and EOD drinking may underlie glial functions with widespread importance in alcohol-related pathology. We present a myriad of novel cell type-specific targets for alcohol dependence that would be difficult to detect using whole tissue analyses. The alcohol-associated glial gene networks presented here, along with their corresponding functional enrichment data, supply a new foundation for defining cellular mechanisms of AUD.

## Supplementary information

Supplementary Information

Table 1

Table 2

## Data Availability

Raw and processed sequencing data from this study have been deposited to the Gene Expression Omnibus under the Accession Number GSE128561.
