# Supplementary material for: Glial gene networks associated with alcohol dependence
Source: Sci Rep. 2019 Jul 29;9:10949. doi: 10.1038/s41598-019-47454-4 (PMC6662804; doi:10.1038/s41598-019-47454-4)
Supplement: Supplementary file 1 — Supplementary Information [file 41598_2019_47454_MOESM1_ESM.docx]

Glial gene networks associated with alcohol dependence

Emma K. Erickson^*^, Yuri A. Blednov, R. Adron Harris, R. Dayne Mayfield

Waggoner Center for Alcohol and Addiction Research, The University of Texas at Austin, Austin, TX, 78712-01095, USA

^*^Corresponding author

**Supplementary Information**


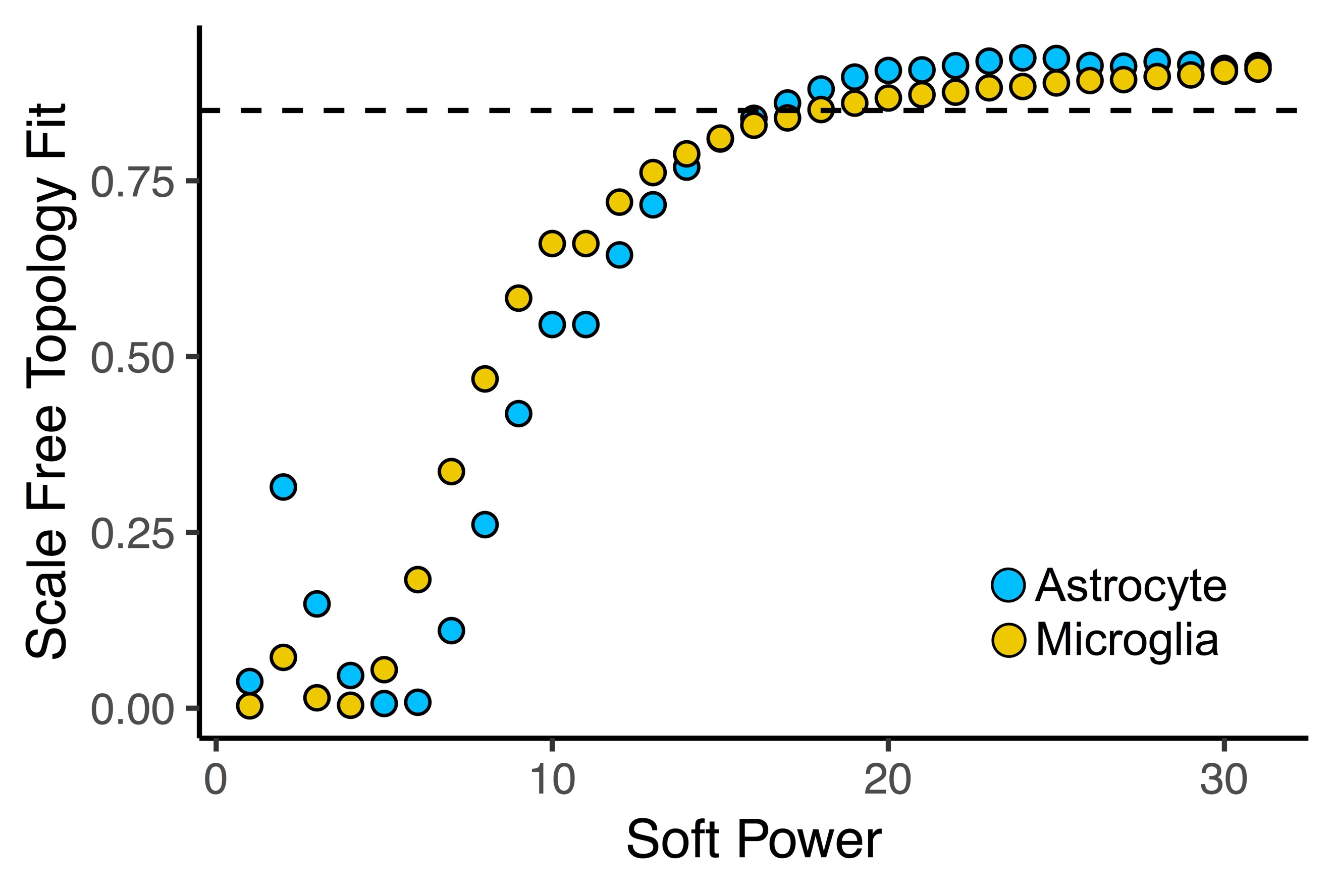


**Supplementary Figure S1**: Scale-free topology plots for astrocyte and microglia gene networks. We used the pickSoftThreshold function to test powers in between 1 and 30 and selected the power (β = 17) that satisfied the fit of scale free topology (indicated by black line at 0.85) in both astrocytes and microglia.


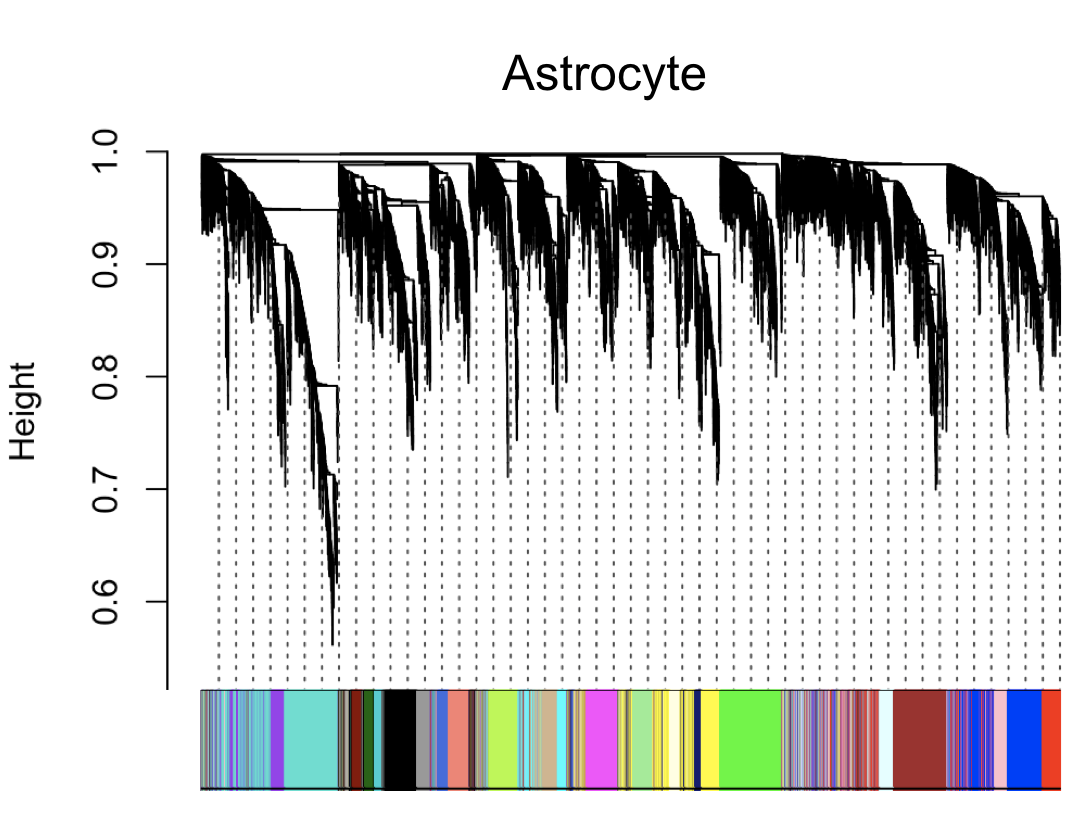

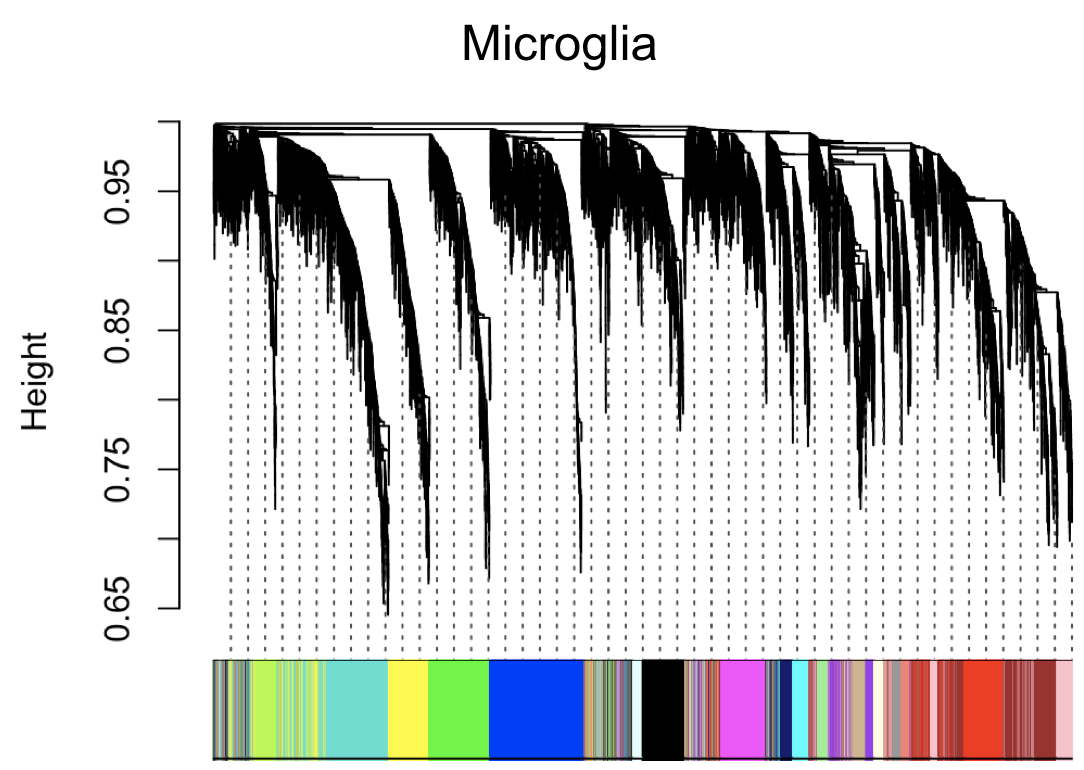


**Supplementary Figure S2**: Gene dendrograms for astrocyte and microglia networks. A dynamic tree-cutting method was used to define modules from branches of genes hierarchically clustered by connection strength.


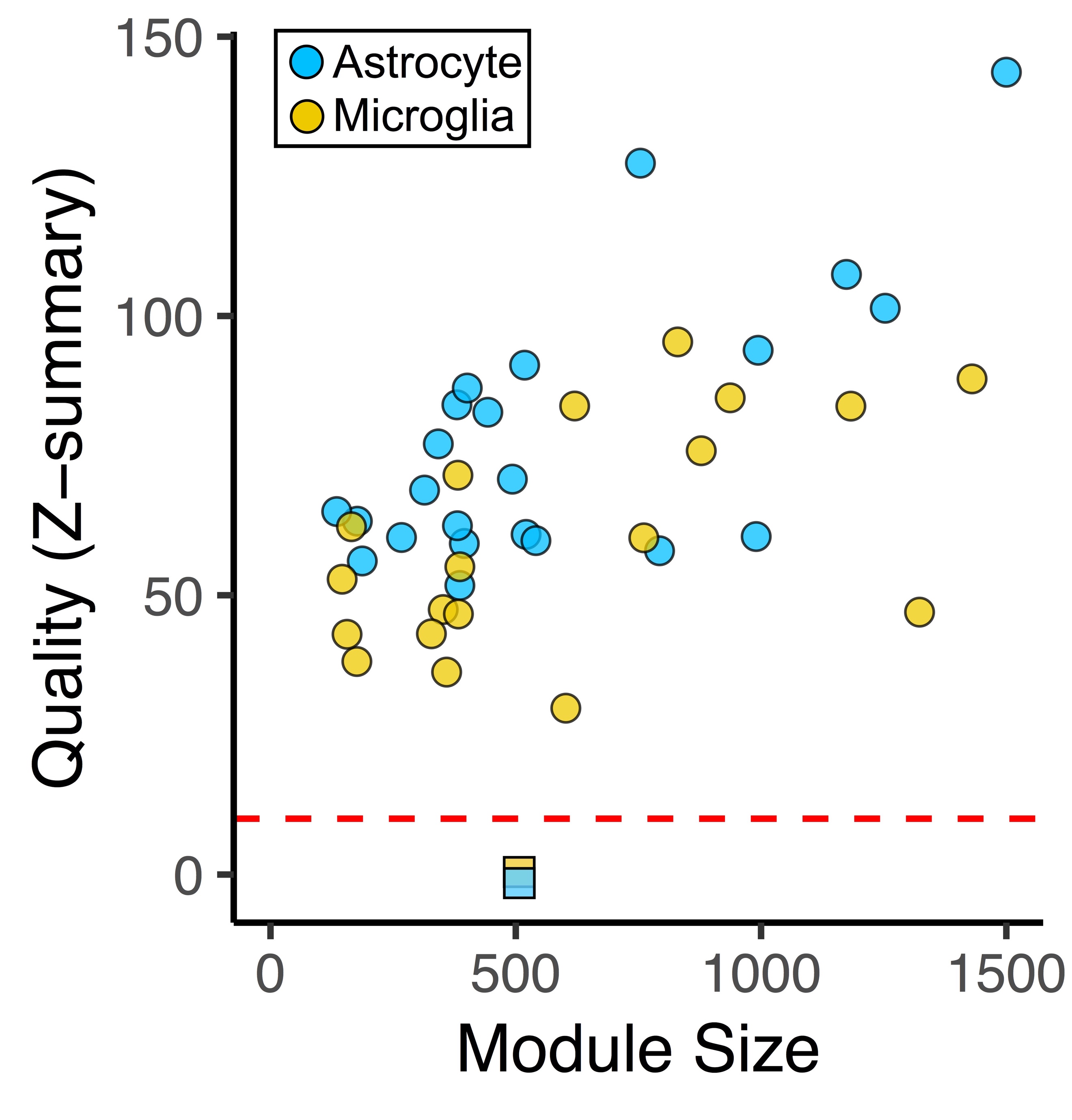


**Supplementary Figure S3**: Module quality assessment for astrocyte and microglia modules. The robustness of gene co-expression modules identified in astrocyte and microglia gene networks was determined using the modulePreservation function. We examined the reproducibility of module assignment in its original dataset by calculating module quality statistics across 100 permutations. The average bootstrapped Z-summary score (y-axis) for each module is shown in the above plot. Squares indicate modules formed by random samplings of network genes. Modules with Z-summary values above 10 are considered well-defined (significance threshold indicated by the red dashed line).
